# A Four-Year Retrospective Study of Amniocentesis in a Tertiary Care Center in South India—Lessons Learnt

**DOI:** 10.1155/jp/9983529

**Published:** 2025-08-21

**Authors:** Jetti Gayatri Jahnavi, Roopa Padavagodu Shivananda, Akhila Vasudeva, Nivedita Hegde, Rashmi Natarajan

**Affiliations:** ^1^Department of Obstetrics and Gynecology, Kasturba Medical College, Manipal, Manipal Academy of Higher Education, Manipal, Karnataka, India; ^2^Division of Fetal Medicine, Department of Obstetrics and Gynecology, Kasturba Medical College, Manipal, Manipal Academy of Higher Education, Manipal, Karnataka, India

**Keywords:** amniocentesis, chromosomal abnormalities, complications, indications, procedure

## Abstract

**Background:** Amniocentesis (AC) remains the most commonly performed prenatal invasive diagnostic test. The data available till now have been collected before the era of high-end ultrasound machines, NIPS, and chromosomal microarrays. In selected cases, whole-exome sequencing is also offered prenatally. The evolution of ultrasound, NIPS, and genetic testing has made us revisit this topic.

**Objective:** We aimed to research and revisit AC data regarding the indications, procedures, genetic testing methods, and outcomes. We reinforce the knowledge of AC, offer tips to minimize complications, and help communicate and counsel patients based on the AC data.

**Methods:** It was a retrospective study from October 2019 to March 2023 in a tertiary care fetal medicine center in a university hospital. A total of 321 patients who underwent AC were analyzed. We observed the demographic details, indications, procedure details, and maternal–fetal and neonatal outcomes.

**Results:** During the study period, 321 patients underwent AC. Abnormal ultrasound findings (71%) were the most common indication for AC. Then, 9% (30/321) had abnormal genetic results. Down syndrome was the most common abnormality (14), followed by Edwards syndrome. Then, 47.96% of cases were in age > 35 years. We had three cases of bloody tap, one leak per vagina, and two missed abortions following AC. Then, 58% had live births.

**Conclusion:** AC is a relatively safe procedure, and even with the advent of NIPS, it remains the gold standard prenatal diagnostic genetic testing method. Major structural anomalies and parental chromosomal anomalies are irreplaceable indications of AC. The technique and expertise of health professionals dictate the complication rate of that center. Chromosomal microarray, DNA storage, and whole-exome sequencing have added an extended armamentarium to our discovery of genetic diseases. Maternal and neonatal outcomes after AC are favorable, so do not hesitate to carry out this invasive test when indicated.

## 1. Background

Amniocentesis (AC) remains the most commonly performed prenatal invasive diagnostic test [1]. It was introduced into clinical practice in the 1970s as the mid-trimester diagnostic investigation of choice for Down syndrome [2]. Here, fetal cells are obtained for cytogenetic analysis.

With the advent of noninvasive prenatal screening (NIPS), except for a few indications, cell-free DNA has taken over, but NIPS remains a screening test [3]. Before taking a major decision of termination of pregnancy based on the NIPS report, it needs to be confirmed by AC. However, in cases of major structural abnormalities on ultrasound, single gene disorders, balanced translocation in the parents, and testing for fetal infections, NIPS is not an option, and direct AC is the gold standard. AC might be an answer where the parents do not want to undergo repeated screening [4].

AC is done from 16 weeks onwards. It is done on an outpatient basis. Under ultrasound guidance (USG), a 20G spinal needle is introduced after discarding an initial 1–2 mL of amniotic fluid to prevent maternal cell contamination (MCC), and 20 mL of amniotic fluid is drawn for genetic testing. Minor risks like maternal discomfort and pain are observed. Major risks include bloody tap, failure to get amniotic fluid, and repeated pricks [2]. Fetal risks include leaking/bleeding/abortion and very rarely fetal injuries. In the hands of an expert, these risks are very rare, and it is a relatively safe procedure.

With technical advances in genetic testing methods, we can now analyze microlevel genes through chromosomal microarray (CMA) and whole-exome sequencing. A wide range of novel genes has been discovered and is still being discovered. Results are obtained quicker, providing the opportunity for parents and health professionals in counseling and providing options for further management of pregnancy [5].

Most of the studies on AC have been done way back. All the data available have been collected before high-end ultrasound machines, NIPS, and recent genetic tests like CMAs. We observed the indications, the procedure, the results, and maternal and fetal/neonatal outcomes after AC in the Division of Fetal Medicine. We proposed practical tips to counsel the mother on the indications and the procedure and provided benchmark data on the rate of complications and tips to reduce complications.

## 2. Materials and Methods

This was a retrospective observational study from October 2019 to May 2023 in a tertiary care center in the Division of Fetal Medicine, Kasturba Hospital, Manipal. All patients either referred or booked at our hospital undergoing anomaly scans at the Division of Fetal Medicine were observed, and those offered AC were studied. Informed consent was taken for the anomaly scans and the AC procedure. The Institutional Ethical Committee (IEC) IEC2-128/2023 approved the study.

### 2.1. Inclusion Criteria

All women who underwent AC were included.

### 2.2. Exclusion Criteria

Women offered AC but deferred were excluded from the study.

### 2.3. Study Protocol

AC was performed with a 20-gauge needle under continuous USG. Initial 1–2 mL of amniotic fluid was discarded, given possible MCC, and 20–30 mL of amniotic fluid was sent for QFPCR, karyotyping, FISH, CMA, or any other specified testing as required. Genetic testing routinely included QFPCR and CMA. In cases of specific phenotypes, likewise, indicated genetic testing was done. Two experienced maternal–fetal medicine experts, having done > 30 procedures, performed the procedure under continuous USG. Both experts were trained in the free-hand technique approach. First, a screening ultrasound to localize the position of the fetus, placental insertion, and cord insertion into the placenta was observed. The needle was inserted into the maximum vertical pocket using a continuous ultrasound technique. All attempts were made to avoid the placenta if feasible. The whole procedure took 10 min. After the AC, the fetal heart rate was checked. In the case of an Rh-negative pregnancy, the mother received 300 *μ*g anti-D immunoglobulin.

The patient was observed for 2 h after AC and was educated about the warning signs; she was asked to report in the presence of warning signs of pain in the abdomen, bleeding, leaking, foul-smelling discharge, and fever. Data on indications, outcomes, and complications of AC were collected from medical records and entered into an Excel sheet. The procedure details and test results were noted. Maternal and fetal outcomes were analyzed.

### 2.4. Outcomes

Obstetrical data included maternal age, parity, and gestational age at the time of AC. Indications like advanced maternal age, abnormal ultrasound findings (included nuchal translucency (NT) ≥ 95^th^ centile, soft markers, and structural anomalies), positive screening for Down syndrome (NIPS/combined screening/quadruple), familial chromosomal diseases, previous pregnancy with fetal anomalies, and previous pregnancy with chromosomal abnormalities were noted. In many, there was more than one indication for AC. We offered AC as the first option when the mother was ≥ 40 years, had major structural anomalies, NT ≥ 99^th^ centile, presence of ≥ 2 soft markers, soft markers like absent nasal bone, and borderline ventriculomegaly, which carry a higher relative risk for aneuploidies and screen positive for aneuploidies. In pregnant ladies aged between 35 and 39, soft markers like echogenic foci in the heart, pelviectasis, or choroid plexus cyst, which have a low relative risk for aneuploidies and screen positive with combined/quadruple/triple testing, who did not want to undergo invasive testing, NIPS was discussed as the first option. However, all the patients were given a general awareness of AC and told it was the final diagnostic test.

Procedural details included gestational age at the time of the procedure, AC done through the placenta, presence of bloody tap, MCC, sampling failure, culture failure, false negative test results if present, and type of genetic testing applied.

The short-term AC-related complications studied included frequency of spontaneous abortion, bleeding, leaking, premature rupture of the membrane, and fetal injuries. Complications within 4 weeks of AC were considered [6]. The results of the AC were studied. Maternal and fetal neonatal outcomes in terms of live birth rates, preterm deliveries, stillbirths, abortion/terminations, and neonatal surgeries, if required, were studied.

### 2.5. Statistical Analysis

Statistical analyses were conducted using the Excel sheet. Descriptive statistics in frequency, mean value, standard deviation (SD), and percentage were used in statistical data processing. Only the frequency of indications was charted for the indications of AC, as there was more than one indication for AC. The abnormal genetic testing results were compared in terms of age and indications with the normal ones.

## 3. Results

**Figure 1:**
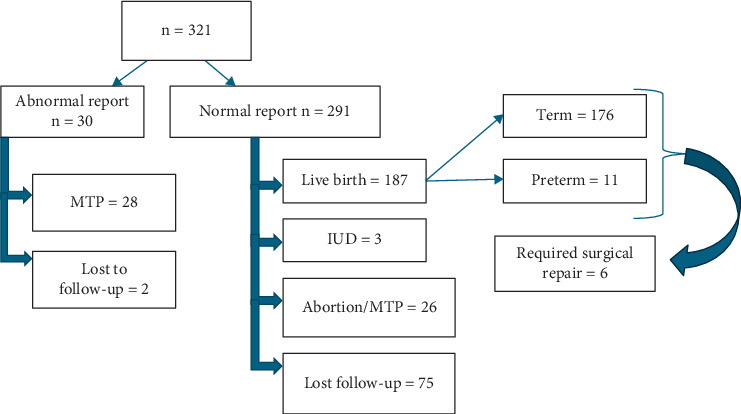
Flow chart of the process of recruitment.

During the study period, 321 patients underwent AC. There were nine cases of double AC for twins.  Figure 1 describes the process of recruitment with the outcome data.

Table 1 depicts the demographic data. Then, 76% of the study population was between 18 and 34 years old. Then, 23% were in the elderly age group, with 4% over 40 years. We had six couples who were consanguineous. When we analyzed age as an association for chromosomal aneuploidies/genetic syndromes, we found that 38.46% (5/13) were in the age group of 40 years. Then, 9.5% (6/63) and 7.7% (19/245) were in the age group of 35–39 and 18–34, respectively.

Table 2 describes the indications for AC. Abnormal ultrasound findings (71%) were the most common indication for AC. High-risk aneuploidy screening (31.8) was the second most common indication. Advanced maternal age contributed to 24% of indications. Please note that a few patients had more than one indication, so the total number exceeded 321. Among the 30 abnormal cases, four had normal USG findings, and AC was performed due to high-risk aneuploidy screening only. The remaining 26 cases had one or the other USG features like thick NT/absent nasal bone, a major structural anomaly, or a combination of the above. One-third of the cases (10/30) had thick NT, and 16% (5/30) had major structural anomalies.

Table 3 narrates the details of the AC procedure. AC was done between 16 and 20 weeks in 86.3%. QFPCR with CMA was the standard genetic testing done. There were three cases of bloody tap and one leak per vagina, and two missed abortions following AC.

**Figure 2:**
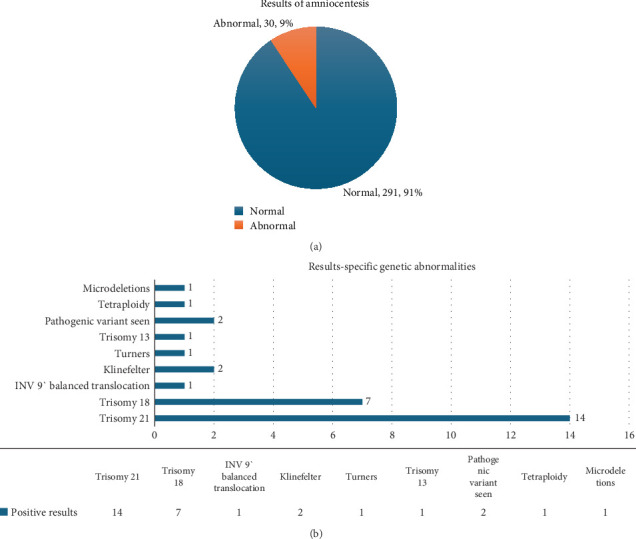
Results of amniocentesis.

Figure 2 describes the genetic analysis of the AC.  Figure 2a describes the percentage of normal and abnormal reports, and Figure 2b the specific genetic abnormality detected. Down syndrome was the most common abnormality (14), followed by Edwards syndrome (seven). The other nine cases were as given in the chart above. Then, 28 underwent termination of pregnancy, and two cases were lost for follow-up.

Two 91 cases, amounting to 90%, had normal reports. Then, 23% were lost to follow-up. Then, 8% underwent termination (spontaneous/induced), and 0.9% had stillbirth.

We had nine cases of twins for double AC. Indications were high risk in aneuploidy screening/thick NT in one twin; one had a tracheoesophageal fistula. The results were normal in all, except in one, which was inconclusive. One underwent spontaneous expulsion, one had preterm delivery, two of the babies had TOF, four were normal, and one was lost to follow-up.

## 4. Discussion

The health of the fetus remains the concern of both the parents and the health professionals [3]. The fetus's structural anatomical assessment is done by ultrasound; however, AC remains the gold standard for genetic diagnosis [7]. ACOG has recommended that AC be offered to all women over 35 [4]; however, in this era of NIPT and the ever-evolving genetic testing on cell-free DNA, the indications of AC need to be reviewed. Though AC is a relatively safe procedure, the associated pregnancy-related loss/leaking/bleeding/infections and very rarely fetal injury [8] need to be reanalyzed in the current setting of high-end ultrasound machines. Even the genetic testing to be sent has evolved from karyotyping to CMA. In selected cases, whole-exome sequencing is also offered prenatally. The evolution of ultrasound, NIPS, and genetic testing has made us revisit this topic.

We aimed to research and revisit AC data regarding the indications, procedures, genetic testing methods, and outcomes; reinforce the knowledge of AC; and offer tips to minimize complications and help communicate and counsel patients.

The indications for diagnostic AC have substantially reduced after the introduction of NIPS. However, ACOG recommends offering AC to all women after 35 years of age and a discussion of all types of screening and invasive testing with pregnant women irrespective of their risk status. ACOG states that every lady has a right to pursue or decline either of the tests [4].

In the study by Grgic et al., maternal age was the most common indication for AC, followed by high-risk screening in 14% and suspicious ultrasound in a mere 4% [6]. Daniilidis et al. quoted 68% of the indications for AC as high-risk for Down syndrome in first-trimester screening [2].

In our study, structural anomalies were the leading indication for AC. Fetal structural anomalies are seen in approximately 3% of pregnancies. Major structural anomalies increase the risk of aneuploidies/genetic syndrome/uniparental disomy, CNVs, and intragenic mutations [9]. Depending on the system involved in USG, the risk of aneuploidies/genetic syndrome also increases. Skeletal, cardiac, and multisystem involvement increases the risk of detecting diagnostic variants in the fetus's genetic testing [9]. In our study, we offered AC to all pregnant ladies with major structural anomalies, regardless of the system involved. Then, 32 cases (9.9%) had NT ≥ 95^th^ centile. Then, 75%–80% of Trisomy 21 fetuses have NT > 95^th^ centile [10]. Thick NT increases the risk of other aneuploidies like Trisomy 18, Trisomy 13, and Turner's syndrome [11].

Combined screening and NIPS were discussed in pregnant ladies with NT between the 95th and 99th centile, and when NT > 99^th^ centile, AC was offered as the first option.

High-risk aneuploidy screening was the second most common indication for AC. Biochemical markers in the quadruple test with maternal age identified 56%–71% of Trisomy 21 pregnancies for a false positive rate of 5%. Maternal age, fetal NT, FHR, and serum-free *β*-hCG and PAPP-A identified 90% of Trisomy 21 pregnancies for a false positive rate of 3% [10, 11]. The triple marker is obsolete, but few of the cases were referred from outside with this test.

In our women, combined screening is universally offered to all women; if this is missed, then quadruple/NIPS are offered. With the decrease in the cost of NIPS and increased awareness and knowledge in pregnant women, many women opt for NIPS as the first screening test. NIPS is a new method for detecting aneuploidies; though still a screening test, it has the highest sensitivity of all the screening tests. It can detect 99% of Trisomy 21 and 98% of Trisomy 18 and 13 with a false positive rate of 0.1%–0.2% [10].

In our study, there were two cases with NIPS positive who underwent AC, with one case positive for Trisomy 21 and underwent termination; the other was negative for Trisomy 21 and continued the pregnancy and delivered a live, healthy baby.

Then, 79 of the 321 cases had maternal age with or without additional risk factors as an indication for AC. The risk of Trisomy 21, 18, and 13 increases with maternal age, whereas Turner and triploidies are unaffected. However, since the number of pregnant women in the younger age group is higher, we see more of Trisomy 21 in the reproductive age group. But, in developed countries, 20% of women have late marriages, and nowadays, 50% of Trisomy 21 are in women with age > 35 years [10].

In our study, 47.96% were either at or above 35 years.

When structural anomalies/thick NT are present, discussion of invasive testing is advised; however, many underwent direct termination without opting for any tests. It is essential to counsel that further testing is offered to prognosticate this and future pregnancies. Though single-step screening is advocated in women who wish to avoid invasives, NIPS can still be offered—however, a positive NIPS warrants AC for confirmation before deciding on continuing or terminating the pregnancy. Earlier studies had maternal age as an indicator for AC, as we knew it increased the risk of aneuploidies. When various aneuploidy screenings were (triple, quadruple, and combined) introduced, they became the major indications for AC. However, with highly sensitive and specific NIPS, maternal age as the only indication for AC is declining, especially between the ages of 35 and 40. However, in women over 40, AC is still their first option. With higher end USG and an increase in the pickup of congenital anomalies, abnormal USG has become a major indication for AC in the present setup.

AC is a relatively safe procedure largely dependent on the technique and expertise of the health professionals. It is possible to perform AC from 11 weeks onwards when it is called early AC. However, for optimal results, it is best done after 15 weeks. AC done before 15 weeks is associated with higher fetal loss, culture failure, and fetal anomalies [12]. In our study, 86% of the cases were between 16 and 20 weeks of gestation. We had four cases after 24 weeks. The legal limit for termination of pregnancy in India is 24 weeks. All these cases reported late to us. One case was Trisomy 18 positive; she delivered vaginally, and the baby died at 8 months of age. The indications in other cases were early-onset severe FGR, nonimmune hydrops, and complex cardiac anomaly, respectively. The genetic testing reports were normal. One had an IUD, the next was lost to follow-up, and the last was delivered. The baby had cardiac surgery and is currently 1–1.5 years old and doing well. We offered AC even after 24 weeks, as the medical board could decide on termination if indicated even after 24 weeks. It helped the couple to prepare for a genetically abnormal baby if they decided to continue the pregnancy, and, as in the last case, it provided reassurance of a good outcome after the baby underwent cardiac repair as the cardiac anomaly was independent of genetic abnormalities. So, late AC done for fetal abnormalities detected late in pregnancy can help in counseling patients, preparing the patients for the care of the newborn, and health professionals to plan the timing and place of delivery [13].

In six cases (1.8%), AC was done through a puncture in the placenta. In all the cases, amniotic fluid was clear, and none had PPROM/abortion. A study done in Japan also shows that AC-related adverse outcomes were not increased following the procedure through the anterior placenta [14]. Though earlier studies showed increased fetal complications [15–17], recent studies refute them [14].

Sampling failure was described as two failed attempts at amniotic fluid aspiration [3]. Our study had 1/321 (0.3%) cases at 16 weeks. She underwent AC for a previous fetus with Turner's syndrome. There was chorion–amnion separation during the screening USG. Still, we proceeded with AC and attempted to retrieve the amniotic fluid in two pricks with different insertion angles at two different sites but failed. We recalled her after 2 weeks when the separation between the chorion and amnion was minimal. We could complete the procedure without further complications. Her AC result was normal, and she had a term delivery. Studies quote that when the separation between amnion and chorion is present, usually before 15 weeks, there is technical difficulty in entry, as in the outer sac, living cells are minimal [3].

Studies quote a sampling failure rate of < 1% [15, 18, 19], and many trials have failed to mention the sampling failure rates [3].

The number of pricks does not increase the rate of fetal loss [16].

Bloody taps were seen in three cases (0.9%). All the bloody taps were altered to greenish-brown, indicating an old bleed. Studies quote a 2% rate of discolored amniotic fluid, indicating intraamniotic bleed. These pregnancies were associated with a higher rate of miscarriages, chromosomal abnormalities, culture failure, and microbial infection [15, 20]. In the presence of altered amniotic fluid, it becomes important to counsel the patient about the possibilities of culture failure, infection, and fetal demise [15].

One of our patients was a 42-year-old with IVF with early fetal demise following 3 weeks of AC. She was hypertensive and diabetic and came with spotting. The reports were negative for aneuploidies and deletion duplication sequence. Two other cases had first-trimester bleeding. One had recurrent pregnancy loss. However, she continued the pregnancy and had a live baby. In the other case, the mother had a balanced Robertsonian translocation, and we could not get culture, but CMA was normal. She continued the pregnancy and delivered a healthy baby who is 2 years old now.

Though care was taken to perform the test with precision, direct entry, and discarding the first 2 mL of fluid, we had one case of MCC; studies estimate an MCC of 0.1%–21% [21–23].

Culture failure was seen in three cases. However, DNA was extracted, and FISH/QFPCR/CMA revealed normal results. Though studies suggest an increased incidence of aneuploidies associated with culture failure, our study failed to show this. Failure to obtain culture was more associated with fetal abnormalities associated with decreased fetal cell shedding [24].

We had one (0.3%) leak per vagina. She was a fourth gravida with one abortion and two preterm deliveries with infant deaths for suspected inborn error of metabolism. She had DCDA twins following a donor egg IVF conception with a cervical stitch. Twin B had esophageal atresia. So, we did double AC with fetal reduction of Twin B. On Day 4 postprocedure, she leaked and developed chorioamnionitis. After cervical stitch removal, she expelled both fetuses. In the absence of an inherent risk for abortion/cervical insufficiency, fluid leak after AC usually has a favorable outcome as the amnion seals off, unlike in this case. Serial monitoring of growth, liquor, and signs of maternal infection are reasonable options [13].

None had fetal injuries in our study. Report on a fetal injury during AC when done under USG is rare [25]. Isolated case reports of direct fetal injury have been documented [26]. However, care must be taken to avoid cord insertion at the anterior placenta and to remove just the amount of amniotic fluid required for genetic testing. Early AC should be avoided as there is an increased risk of talipes [27].

Then, 49% of the patients underwent karyotyping, our institute's most common genetic test till 2021. In 2021, CMA was introduced as our routine testing. Then, 48% underwent CMA. If aneuploidies and deletion-duplication turned negative in rare phenotypes, we proceeded with whole-exome sequencing with the stored DNA sample. Then, 30 out of 321 cases had genetic variants, contributing to 9% of genetic abnormalities in this population. Then, 27 cases had genetic abnormalities detected by karyotype, and 3/30, amounting to 10% of the cases, had additional deletion and duplication picked up by CMA. Adding CMA to genetic testing increased the detection of genetic abnormalities by 3%–5% [28].

Down syndrome was the most common abnormality in our study, amounting to 46.6% (14/30). Then, 23% (7/30) was Edwards syndrome. Grgic et al. reported Down syndrome to be the most common abnormality, followed by Edwards syndrome, similar to our study. However, the percentage reported was smaller, 26% and 6.8%, respectively, compared to 46% and 23% in our study [6].

Prenatal whole-genome sequencing has been described purely on a research basis. However, whole-exome sequencing and targeted gene testing are widely used in clinical practice due to lower cost, rapid turnover time, greater sequencing depth, and smaller amount of DNA required [9].

WES can identify a genetic cause in 10% of the fetuses in whom karyotype and CMA were normal. Gene variants related to developmental disorders were shown in > 50% of the fetuses studied [9].

We had one case where we had AC for ventriculomegaly with craniosynostosis, and the CMA came normal. However, we had stored the DNA. We offered whole-genome testing when she came to us in the subsequent pregnancy at 6 weeks. The result was Munkes syndrome. We tested the parents for the gene, and the husband was positive. But he had a normal phenotype. AC in this pregnancy revealed the fetus to be positive. However, the couple continued the pregnancy, and the neurosonogram to date was normal. In the case of unique/rare structural anomalies, counsel the patient to store the DNA. WGS can be done in interpregnancy/early pregnancy so that the management of this pregnancy can be planned.

Then, 58% had live births. Daniilidis et al. report a similar outcome in their study [2].

There were three intrauterine deaths. Two were low-risk primigravida; one fetus developed hydrops at 7 months, and the other had severe FGR at 31 weeks. In the last multigravida, the cause was not known. In all the above cases, the genetic testing report was normal.

Six cases required some form of treatment for major structural anomalies. One case had left nephroureterectomy with left orchidopexy, two cases of CTEV had cast application, one case of left ectopic kidney is on follow-up, and the last two had VSD, for which both underwent VSD closure. One of the babies had vaginal synechiae release also. All these babies are doing well. The normal results in AC, despite the major structural anomalies, reassured the couple and gave them the confidence to continue the pregnancy and plan for the treatment of the newborn.

## 5. Conclusion

AC is a relatively safe procedure, and even with the advent of NIPS, it remains the gold standard prenatal diagnostic genetic testing method. Major structural anomalies and parental chromosomal anomalies are irreplaceable indications of AC. The technique and expertise of health professionals dictate the complication rate of that center. CMA, DNA storage, and whole-exome sequencing have added an extended armamentarium to our discovery of genetic diseases. Maternal and neonatal outcomes after AC are favorable, so do not hesitate to carry out this invasive test when indicated.

## Figures and Tables

**Table 1:** Demographic data of the pregnant women who underwent amniocentesis.

**Demographic data**	**Total number of cases, ** **n** = 321** (%)**
Age	
18–34	245 (76.3)
35–39	63 (19.6)
≥ 40	13 (4.2)
Parity	
Primigravida	130 (40)
Multigravida	191 (60)
Consanguineous marriage	6 (1.8)

**Table 2:** Indications for amniocentesis.

**Indications for amniocentesis**	**Number**
Advanced maternal age > 35	79 (24)
35–39	66
≥ 40	13
High-risk aneuploidy screen	102 (31.8)
NIPS	02 (0.01)
Dual test	87 (27.7)
Triple test	08 (2.5)
Quad test	05 (1.5)
Previous history of aneuploidy	03
No report in CVS	01
Abnormal anomaly scan (early/late)	226 (71.2)
1. Structural anomalies	41
2. Abnormal NT (> 95^th^ centile)	32 (10)
95^th^–98^th^	17
≥ 99^th^	15
3. ≥ 2 soft markers	52
4. Single soft markers	47

*Note:* Note that few patients had more than one indication, so the numbers exceeded 321.

**Table 3:** Details on the technique of amniocentesis.

**Details on the technique of amniocentesis**	**n** = 321** (%)**
Gestational age at procedure	
16–20 weeks	275 (86.3)
21–24 weeks	42 (13)
> 24 weeks	4 (1.2)
Puncture through the placenta	6 (1.8)
Bloody tap	3 (0.9)
Maternal cell contamination	1
Sampling failure	1 (0.3)
Culture failure	3 (0.9)
False-negative	0
Genetic testing	
Karyotyping	18 (5.6)
Karyotyping + QFPCR	138 (43.5)
CMA + QFPCR	140 (44.1)
CMA + QFPCR + karyotype	13 (4.1)
FISH	8 (2.5)
Complications following the procedure	
Leaking	1 (0.3)
Missed abortion	2 (0.6)

## Data Availability

The data that support the findings of this study are available from the corresponding author upon reasonable request.
